# Experimentally probing anomalous time evolution of a single photon

**DOI:** 10.1093/pnasnexus/pgad157

**Published:** 2023-05-11

**Authors:** Ryo Okamoto, Eliahu Cohen

**Affiliations:** Department of Electronic Science and Engineering, Kyoto University, Kyoto Daigaku-Katsura, Nishikyo-ku, 615-8510 Kyoto, Japan; PRESTO, Japan Science and Technology Agency, 4-1-8 Honcho, Kawaguchi, 332-0012 Saitama, Japan; Faculty of Engineering and Institute of Nanotechnology and Advanced Materials, Bar Ilan University, 5290002 Ramat Gan, Israel

**Keywords:** quantum dynamics, sequential weak value, heralded single photon, quantum optics

## Abstract

In quantum mechanics, a quantum system is irreversibly collapsed by a projective measurement. Hence, delicately probing the time evolution of a quantum system holds the key to understanding curious phenomena. Here, we experimentally explore an anomalous time evolution, where, illustratively, a particle disappears from a box and emerges in a different box, with a certain moment in which it can be found in neither of them. In this experiment, we directly probe this curious time evolution of a single photon by measuring up to triple-operator sequential weak values (SWVs) using a novel probeless scheme. The naive interpretation provided by single-operator weak values (WVs) seems to imply the “disappearance” and “re-appearance” of a photon as theoretically predicted. However, double- and triple-operator SWVs, representing temporal correlations between the aforementioned values, show that spatial information about the photon does not entirely vanish in the intermediate time. These results show that local values (in space and time) alone, such as single-operator WVs, cannot fully explain all types of quantum evolution in time—higher order correlations are necessary in general, shedding new light on time evolution in quantum mechanics. The probeless measurement technique proposed here for measuring multiple-operator WVs can be straightforwardly extended to study various other cases of curious quantum evolution in time.

**Significance Statement**

Quantum dynamics is remarkable, but probing it often distorts its subtle features. Here, we experimentally explore a counterintuitive time evolution of a single photon in which the photon seems to leave a box, where it previously resided, and later re-appears in a different box. We show that this apparent paradox is relaxed once higher order temporal correlations are considered and conclude that local values (in space and time) are insufficient, in general, for fully describing quantum dynamics. This result sheds new light on time evolution in quantum mechanics and the unique temporal correlations it entails.

## Introduction

Probing and analyzing the time evolution of a dynamical system plays an essential role in physics. In classical physics, the time evolution of physical quantities can be precisely measured without disturbing the system. In contrast, the time evolution in quantum mechanics is often irreversibly perturbed by the back-action of measurement and the apparent collapse of the wavefunction. However, this limitation can be relaxed to some extent when considering weak measurements in which some information on an observable can be extracted at each time as a weak value (WV) (1, 2). Weak measurements have thus opened up new ways to explore time evolution in quantum physics, which has enabled, for instance, average Bohmian trajectories of photons in a double-slit experiment (3, 4) and nonclassical trajectories of a superconducting qubit (5, 6) to be revealed, or the past history of quantum particles to be explored (7, 8). Sequential weak values (SWVs), which are sensitive to the dynamics of the system (9), have been experimentally demonstrated in recent years (10) and have attracted attention as a new measurement technique that has allowed direct measurements of quantum states (11) and processes (12), as well as measurements of noncommuting observables (10, 13). Furthermore, SWVs directly correspond to the probability amplitudes along virtual Feynman histories when the observables are projective operators (14).

Recently, a theoretical proposal of an anomalous time evolution of a particle has been presented by Aharonov et al. (15). In this proposal, a particle is prepared (preselected) in a superposition state over three boxes and is postselected in a particular state. Moreover, the particle has nontrivial dynamics, which enables it to tunnel between the first and second boxes. Aharonov et al. have shown that during its time evolution, the particle can be previously found with certainty in the first box, “disappears” from both the first and second boxes, and then “re-appears” in the second box.

In this article, we experimentally demonstrate and analyze this paradoxical time evolution using SWV measurements. Our experimental implementation encodes the original paradox involving matter waves, within a quantum optical setup. It is based on single-photon propagation in a three-mode interferometer where three optical modes correspond to the three boxes described in the original paradox. We have developed a novel probeless scheme to measure the SWVs for arbitrary time series. Unlike the original proposal in ref. (1), this scheme does not require a probe, such as another degree of freedom or another photon, which makes it very suitable for the exploration of complex quantum dynamics. The naive interpretation provided by the observed single-operator WVs seems to indicate the disappearance of a photon from both the first and second modes and the re-appearance of the photon in the second mode, thereby demonstrating the disappearing and re-appearing particle (DRP) scenario. However, the double- and triple-operator SWVs show that the photon passes through both the first and second modes in the intermediate time. This subtle interplay between single- and multioperator WVs shows their complementary nature and the importance of both in describing the past history of quantum systems.

## Theoretical description

**Fig. 1.**
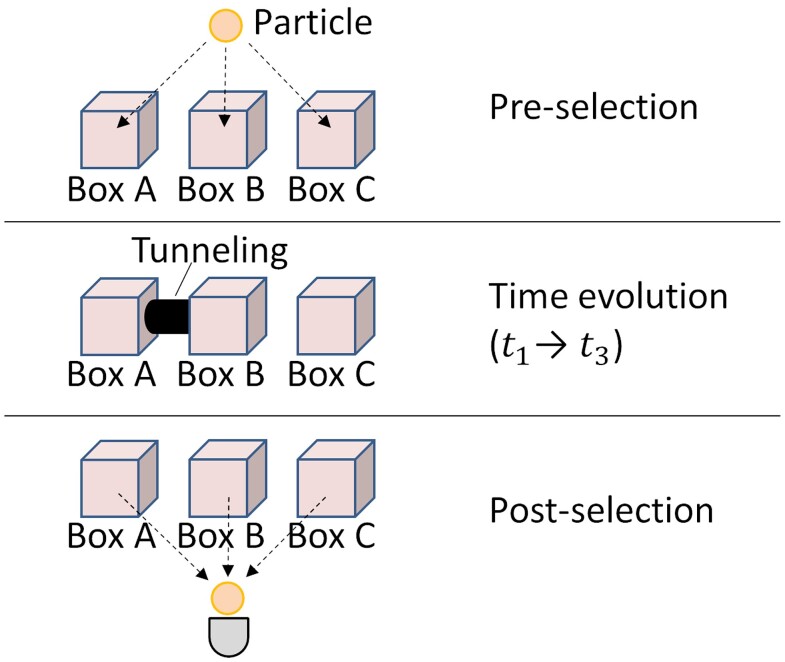
Schematic illustration of DRP. First, a particle is preselected in the state shown in Eq. 1. Then, Boxes A and B are connected by a narrow passage that allows tunneling between them. Finally, the particle is postselected in the state shown in Eq. 3.

Fig. 1 shows a schematic illustration of the time evolution of a DRP. First, a particle is preselected in the superposition state:


(1) $$\left|\psi_{i}\right\rangle =\frac{1}{\sqrt{3}}(-i\left|A\right\rangle -\left|B\right\rangle +\left|C\right\rangle ),$$

where |A⟩, |B⟩, and |C⟩ correspond to the states where the particle occupies Boxes A, B, and C, respectively, and ⟨A|B⟩=⟨A|C⟩=⟨B|C⟩=0. Then, Boxes A and B are connected by a narrow passage that allows tunneling between them. The tunneling begins at time $t_{1}$ and continues until time $t_{3}$. We also consider time $t_{2}$, which is exactly half the time between $t_{1}$ and $t_{3}$. The unitary transformations from $t_{1}$ to $t_{2}$ and from $t_{2}$ to $t_{3}$ are both given by the following matrix representation:


(2) $$\hat{U}=\left(\begin{array}{ccc} \frac{1}{\sqrt{2}} & -\frac{i}{\sqrt{2}} & 0\\ -\frac{i}{\sqrt{2}} & \frac{1}{\sqrt{2}} & 0\\ 0 & 0 & 1 \end{array}\right),$$

where $\left|A\right\rangle =(1,0,0{)}^{T}$, $\left|B\right\rangle =(0,1,0{)}^{T}$, and $\left|C\right\rangle =(0,0,1{)}^{T}$. Consequently, the preselected state evolves to ${\hat{U}}^{2}\left|\psi_{i}\right\rangle$ at $t_{3}$. At $t_{3}$, the state is postselected in the superposition state.


(3) $$\left|\psi_{f}\right\rangle =\frac{1}{\sqrt{3}}(-\;i\left|A\right\rangle -\left|B\right\rangle +\left|C\right\rangle ).$$

Through the tunneling process, the postselected state can evolve backwards in time. The backward time evolution from $t_{3}$ to $t_{2}$ and from $t_{2}$ to $t_{1}$ are both represented by ${\hat{U}}^{†}$.

In the DRP, the particle in Box A at $t_{1}$ seems to disappear from both Boxes A and B at $t_{2}$ and then seems to re-appear in Box B at $t_{3}$ (15). These predictions can be individually verified by projective measurements at the corresponding times (15) or by strong measurements using a probe photon (16, 17). Such unusual time evolution is also supported by WVs. The WV for the occupation of Box A by the particle at $t_{1}$ is given by $\langle \hat{A}(t_{1})\rangle_{w}=\left\langle \psi_{f}\right|({\hat{U}}^{†}{)}^{2}\hat{A}\left|\psi_{i}\right\rangle /\left\langle \psi_{f}\right|{\hat{U}}^{2}\left|\psi_{i}\right\rangle =1$, where $\hat{A}\equiv \left|A\right\rangle \left\langle A\right|$, showing the presence of the particle in Box A. When the system evolves to $t_{2}$, $\langle \hat{A}(t_{2})\rangle_{w}=\left\langle \psi_{f}\right|{\hat{U}}^{†}\hat{A}\hat{U}\left|\psi_{i}\right\rangle /\left\langle \psi_{f}\right|{\hat{U}}^{2}\left|\psi_{i}\right\rangle =0$ and $\langle \hat{B}(t_{2})\rangle_{w}=\left\langle \psi_{f}\right|{\hat{U}}^{†}\hat{B}\hat{U}\left|\psi_{i}\right\rangle /\left\langle \psi_{f}\right|{\hat{U}}^{2}\left|\psi_{i}\right\rangle =0$ shows that the particle seems to disappear from Boxes A and B. At $t_{3}$, $\langle \hat{B}(t_{3})\rangle_{w}=\left\langle \psi_{f}\right|\hat{B}{\hat{U}}^{2}\left|\psi_{i}\right\rangle /\left\langle \psi_{f}\right|{\hat{U}}^{2}\left|\psi_{i}\right\rangle =1$, which indicates that the particle seems to re-appear in Box B.

## Experimental setup

**Fig. 2.**
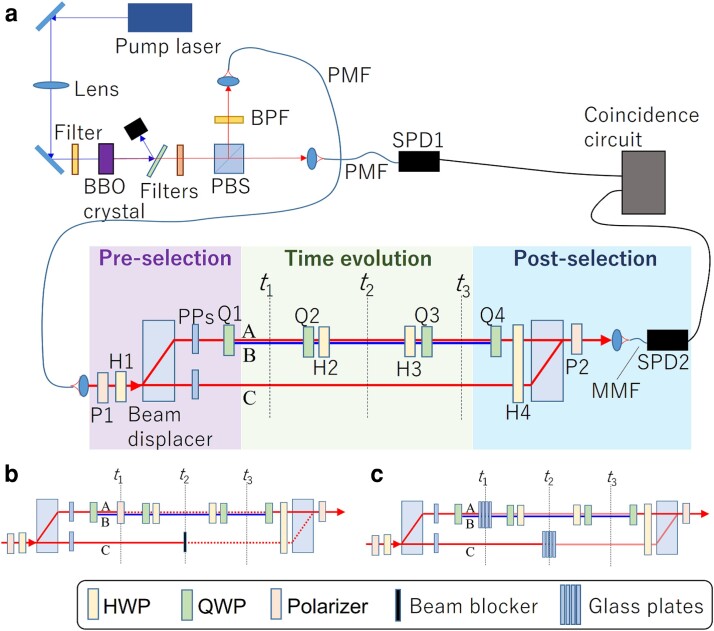
a) Schematic of the experimental setup. b, c) Three-mode interferometer part with attenuators required for measuring an SWV of $\langle \hat{A}(t_{1})\hat{C}(t_{2})\rangle_{w}$. BBO, β-barium borate; PBS, polarizing beam splitter; BPF, band pass filer; PMF, polarization maintaining fiber; SPD, single-photon detector; PP, phase plate; MMF, multimode fiber; HWP, half-wave plate; QWP, quarter-wave plate.

To explore this curious time evolution, we use a highly stable three-mode interferometer with a single-photon input, as shown in Fig. 2a. Single photons with a wavelength of 808 nm are generated from a heralded single-photon source, consisting of a continuous wave (cw) diode pump laser (404 nm) and a 4-mm-long β-barium borate (BBO) crystal cut for Type II phase matching. The pairs of photons are generated collinearly with the pump beam via a parametric down-conversion process. One of the photons in the pair is horizontally polarized and the other is vertically polarized. The pump beam is removed from the pairs of photons using filters. The pairs of photons are separated at a polarizing beam splitter; one photon from each pair is detected as a trigger by a single-photon detector (SPD1), while the other photon is guided to the setup for DRP time evolution after passing through a 4-nm band pass filter centered at 808 nm which serves to remove any unwanted background light. In the first step of the DRP time evolution, the polarization of the photons is purified using a polarizer (P1) and the preselected quantum state $\left|\psi_{i}\right\rangle$ is then prepared using a beam displacer and a quarter-wave plate (Q1). Boxes A and B are implemented as the horizontal and vertical polarization modes, respectively. The beam displacer is used to create the spatial mode corresponding to Box C. Two phase plates are used to control the phase and optical path lengths. Tunneling between Boxes A and B that corresponds to $\hat{U}$ is effectively implemented using a quarter-wave plate (Q2) and a half-wave plate (H2), which transforms the quantum state into $\hat{U}\left|\psi_{i}\right\rangle$. The quantum state similarly evolves to ${\hat{U}}^{2}\left|\psi_{i}\right\rangle$ after the second tunneling effect implemented by a half-wave plate (H3) and a quarter-wave plate (Q3). The required postselection $\left|\psi_{f}\right\rangle$ is realized using a quarter-wave plate (Q4), a half waveplate (H4), the second beam displacer, and a polarizer (P2). The photons are then sent to a single-photon detector (SPD2). The coincidence circuit counts the number of heralded single photons that arrive at the output.

## Measurement of SWVs

So far, the transverse displacement of a photon has been widely used as a measurement pointer for weak measurement (18) and has been extended to sequential weak measurements (10). However, it is restricted to measurement up to double-operator SWVs because of the limited degrees of transverse freedom. Although a different approach enables weak measurement to be implemented for an arbitrary number of multiple-operator SWVs (13), it is designed for polarization measurements and requires concatenated interferometers.

As an alternative approach, a probeless method has been proposed and demonstrated with neutrons (19). In this method, the WV appears in the change of the postselection probability amplitude when a small attenuation is given between the pre- and postselections. Recently, this method has been theoretically generalized, for example, so as to be applicable to a quantum system in mixed states (20). However, the way to measure SWVs was not presented and the degree of attenuation was restricted to be weak.

We extend the probeless method to make arbitrary degree of attenuation available and also develop a way to extract SWVs, making it experimentally feasible and convenient for the exploration of complicated quantum dynamics. The following is a description of the procedure of our probeless scheme, including an example for measuring an SWV of $\langle \hat{A}(t_{1})\hat{C}(t_{2})\rangle_{w}$, which corresponds to the sequential occupation of Mode A at $t_{1}$ and Mode C at $t_{2}$. In our scheme, the process of finding a WV or SWV is almost equivalent to solving a system of equations with two unknown values which correspond to the real and imaginary parts of a WV or SWV (see Materials and Methods for details). Since two equations are required to identify the two unknown values, two measurements with two different sets of attenuators are performed, one each in Steps 2 and 3 of the procedure below.

1. The number of heralded single photons *N* at the output of the three-mode interferometer shown in Fig. 2a is counted.
2. A set of attenuators is placed and the number of heralded single photons $N_{L1}$ at the output is counted. Each attenuator is inserted into a target mode (A, B, or C) at a target time step ($t_{1}$, $t_{2}$, or $t_{3}$). For each attenuator, we adopted an attenuation of 100% that was simply realized by blocking a mode: a polarizer was used for Modes A and B, and a beam blocker was used for Mode C. In the case of measuring $\langle \hat{A}(t_{1})\hat{C}(t_{2})\rangle_{w}$, attenuators are inserted into Mode A at $t_{1}$ and Mode C at $t_{2}$ as shown in Fig. 2b.
3. The attenuators are replaced with those having different degree of attenuation and the number of heralded single photons $N_{L2}$ at the output is counted. For the different degree of attenuation, a moderate attenuation of approximately 70% (30% in transmittance) was adopted in the experiment, which was realized using a set of four Brewster-angle glass plates to yield a polarization-dependent moderate attenuation. Fig. 2c shows the setup with the attenuators for measuring $\langle \hat{A}(t_{1})\hat{C}(t_{2})\rangle_{w}$.
4. A WV or SWV is estimated from *N*, $N_{L1}$ and $N_{L2}$ obtained in the above steps. For instance, to estimate $\langle \hat{A}(t_{1})\hat{C}(t_{2})\rangle_{w}$, Eq. 10 is minimized under the conditions of Eq. 11 (see Materials and Methods for theoretical details). The values of $N_{L1}/N$ and $N_{L2}/N$ are substituted into $p_{\mathrm{DL}1}/p$ and $p_{\mathrm{DL}2}/p$ in Eq. 11, respectively. To obtain *X* in Eq. 11, the local WVs corresponding to $\langle \hat{A}(t_{1})\hat{C}(t_{2})\rangle_{w}$, i.e. $\langle \hat{A}(t_{1})\rangle_{w}$ and $\langle \hat{C}(t_{2})\rangle_{w}$, must be measured beforehand using the same procedure. The other parameters in Eq. 11, $\gamma_{k1}$, $\gamma_{l1}$, $\gamma_{k2}$, and $\gamma_{l2}$, can be precisely estimated through the transmittance measurement of the attenuators used in Steps 2 and 3.

## Results and discussion

**Fig. 3.**
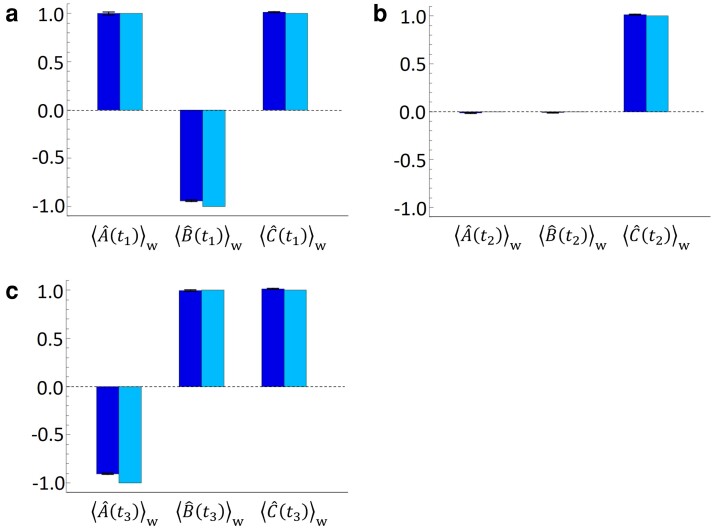
Single-operator WVs at $t_{1}$, $t_{2}$, and $t_{3}$ are shown in panels a), b), and c), respectively. The WV for Mode C is not dependent on time; therefore, the same experimental value is shown for $\langle \hat{C}(t_{1})\rangle_{w}$, $\langle \hat{C}(t_{2})\rangle_{w}$, and $\langle \hat{C}(t_{3})\rangle_{w}$. The left and right bars indicate experimental and theoretical results, respectively. Error bars correspond to the standard error taking into account the uncertainty of the related quantities.

In the experiment, the single-operator WVs were measured first; the estimated single-operator WVs are shown in Fig. 3. The left and right bars indicate the experimental results and the theoretical predictions, respectively. Note that the imaginary parts of the estimated WVs are not shown, because their magnitudes, which are zero in theory, are found to be almost zero within the error. The observed WV for Mode A at $t_{1}$ is $\langle \hat{A}(t_{1})\rangle_{w}=0.999\pm 0.016$, which shows that the photon is almost certainly in Mode A at $t_{1}$. For Mode B, a negative WV was observed as $\langle \hat{B}(t_{1})\rangle_{w}=-0.940\pm 0.007$, which lies outside of the range of eigenvalues for projection operators. However, the WV for Mode C at $t_{1}$ is also almost 1 as $\langle \hat{C}(t_{1})\rangle_{w}=1.014\pm 0.004$; therefore, the sum of all the WVs at $t_{1}$ becomes close to 1, as for usual probability behavior: $\langle \hat{A}(t_{1})\rangle_{w}+\langle \hat{B}(t_{1})\rangle_{w}+\langle \hat{C}(t_{1})\rangle_{w}=1.07\pm 0.02$. At $t_{2}$, as predicted by the DRP thought experiment (15), the photon seems to disappear from Modes A and B because the WVs for Modes A and B are very close to zero: $\langle \hat{A}(t_{2})\rangle_{w}=-0.014\pm 0.004$ and $\langle \hat{B}(t_{2})\rangle_{w}=-0.009\pm 0.005$. At $t_{3}$, the photon seems to re-appear in Mode B because of $\langle \hat{B}(t_{3})\rangle_{w}=0.996\pm 0.007$, while the WV for Mode A is close to −1 as $\langle \hat{A}(t_{3})\rangle_{w}=-0.905\pm 0.007$. The theoretical predictions (right bars) are consistent with the experimental results. The slight deviation from the theoretical results mainly originates from the imperfection in the three-mode interference.

The observed negative WVs of $\langle \hat{B}(t_{1})\rangle_{w}=\langle \hat{A}(t_{3})\rangle_{w}≃-1$ can be interpreted in several ways. In ref. (21), negative WVs were understood as “negative probabilities” that arise in counterfactual scenarios, while in ref. (22), they implied the negation of operations. From a complementary perspective, the negative WVs were understood as negative properties of particles within pre- and postselected systems (23) that contribute to the construction of the so-called “weak reality” (24, 25), i.e. the quantum reality between two projective measurements as evidenced by intermediate weak measurements. In this sense, the negative WVs may describe the “particle–counterparticle” dynamics within the weak reality, i.e. at times $t_{1}<t<t_{3}$, the negative and positive WVs tunnel through the boxes and start to mix, until time $t_{2}$ when they completely cancel each other. Therefore, the sum of WVs within Boxes A and B is zero throughout the time evolution, which provides a self-consistent particle-like description of the system.

**Fig. 4.**
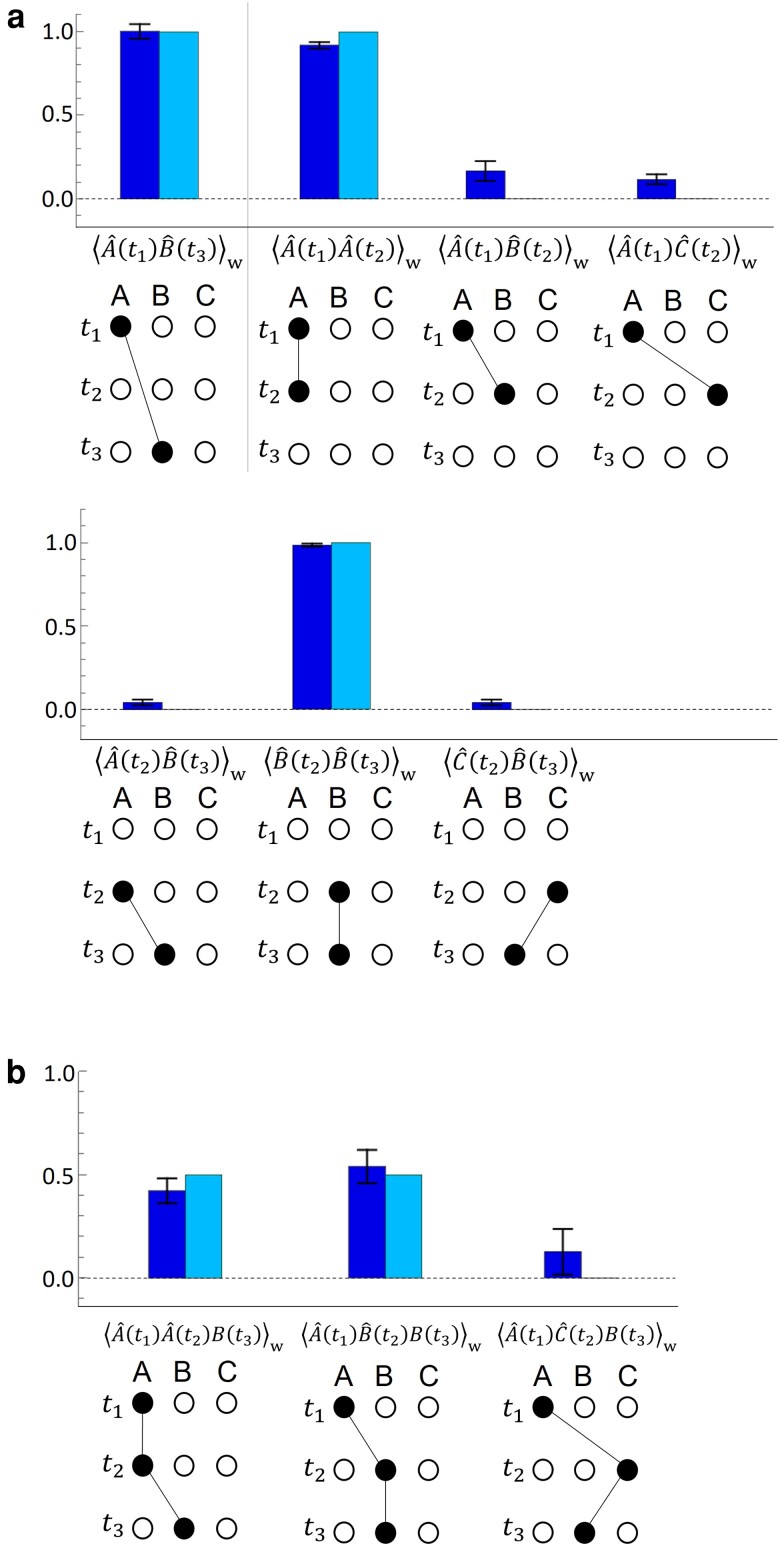
Double- and triple-operator SWVs are described in panels a) and b), respectively. The left and right bars indicate experimental and theoretical results, respectively. Error bars correspond to the standard error taking into account the uncertainty of the related quantities. The black lines that connect the black dots visually show the naive path that corresponds to each SWV.

The double-operator SWVs were then evaluated, which correspond to the sequential occupation of two boxes at two different times. For instance, the double-operator SWV for the sequential occupation of Mode A at $t_{1}$ and Mode B at $t_{2}$ can be written as $\langle \hat{A}(t_{1})\hat{B}(t_{2})\rangle_{w}\equiv \left\langle \psi_{f}\right|\hat{U}\hat{B}\hat{U}\hat{A}\left|\psi_{i}\right\rangle /\left\langle \psi_{f}\right|\hat{U}\hat{U}\left|\psi_{i}\right\rangle$. Fig. 4a shows the estimated double-operator SWVs. The obtained SWV of $\langle \hat{A}(t_{1})\hat{B}(t_{3})\rangle_{w}=1.00\pm 0.04$ indicates that a photon starting in Mode A at time $t_{1}$ will move with certainty to Mode B at time $t_{3}$ under the current Hamiltonian, as could be expected. Single-operator WVs also support this intuitive story, although there are some other and possibly more interesting SWVs to explore. $\langle \hat{A}(t_{2})\rangle_{w}=-0.014\pm 0.004$ may intuitively predict $\langle \hat{A}(t_{1})\hat{A}(t_{2})\rangle_{w}∼0$; however, $\langle \hat{A}(t_{1})\hat{A}(t_{2})\rangle_{w}$ is 0.92±0.02, which indicates that the photon does not simply disappear at $t_{2}$. These results suggest that local values, such as single-operator WVs, may not be able to fully explain the richness of the quantum time evolution. However, such a single-operator description of the nontrivial distribution of the photon can be augmented using SWVs.

Another SWV of $\langle \hat{B}(t_{2})\hat{B}(t_{3})\rangle_{w}=0.98\pm 0.01$ also indicates that the photon does not disappear at $t_{2}$, but rather it shows that a photon may choose a path of Mode B → Mode B from $t_{2}$ to $t_{3}$, which presents an apparent discontinuity in the light path Mode A → Mode A that the photon took between $t_{1}$ and $t_{2}$. This discontinuity of the observed SWVs is attributed to the lack of overlap between the preselected state and the postselected state in the subspace that consists of Modes A and B at $t_{2}$: $\hat{U}\left|\psi_{i}\right\rangle =(-\sqrt{2}\left|B\right\rangle +\left|C\right\rangle )/\sqrt{3}$ and ${\hat{U}}^{†}\left|\psi_{f}\right\rangle =(-\sqrt{2}i\left|A\right\rangle +\left|C\right\rangle )/\sqrt{3}$, which is the reason why a photon seems to disappear at $t_{2}$ according to the single-operator WVs.

The linearity rule that the SWV follows (9) can be used to find a relation that connects the single- and double-operator SWVs as follows: $\langle \hat{A}(t_{1})\hat{A}(t_{2})\rangle_{w}+\langle \hat{A}(t_{1})\hat{B}(t_{2})\rangle_{w}+\langle \hat{A}(t_{1})\hat{C}(t_{2})\rangle_{w}=\langle \hat{A}(t_{1})(\hat{A}(t_{2})\;+\hat{B}(t_{2})+\hat{C}(t_{2}))\rangle_{w}=\langle \hat{A}(t_{1})\hat{I}\rangle_{w}=\langle \hat{A}(t_{1})\rangle_{w}$. We observe that $\langle \hat{A}(t_{1})\hat{A}(t_{2})\rangle_{w}+\langle \hat{A}(t_{1})\hat{B}(t_{2})\rangle_{w}+\langle \hat{A}(t_{1})\hat{C}(t_{2})\rangle_{w}=1.20\pm 0.07$, which is almost in agreement with the theoretical prediction ($\langle \hat{A}(t_{1})\rangle_{w}=1$), and the experimental value ($\langle \hat{A}(t_{1})\rangle_{w}=0.999\;\pm 0.016$). Similarly, $\langle \hat{A}(t_{2})\hat{B}(t_{3})\rangle_{w}+\langle \hat{B}(t_{2})\hat{B}(t_{3})\rangle_{w}+\langle \hat{C}(t_{2})\hat{B}(t_{3})\rangle_{w}\;=\langle \hat{B}(t_{3})\rangle_{w}$ can be obtained. The experimental results show that $\langle \hat{A}(t_{2})\hat{B}(t_{3})\rangle_{w}+\langle \hat{B}(t_{2})\hat{B}(t_{3})\rangle_{w}+\langle \hat{C}(t_{2})\hat{B}(t_{3})\rangle_{w}=1.06\pm 0.03$, which is in agreement with the theoretical prediction ($\langle \hat{B}(t_{3})\rangle_{w}=1$) and the experimental value ($\langle \hat{B}(t_{3})\rangle_{w}=\;0.996\pm 0.007$).

Finally, the triple-operator SWVs were measured. Fig. 4b shows the estimated triple-operator SWVs. The experimental results again show that the photon does not disappear at $t_{2}$. It is also shown that the photon avoids the path Mode A → Mode C → Mode B, which can be naively predicted by the single-operator WVs shown in Fig. 3. The obtained value of $\langle \hat{A}(t_{1})\hat{A}(t_{2})\hat{B}(t_{3})\rangle_{w}$ is 0.42±0.06, although $\langle \hat{A}(t_{2})\hat{B}(t_{3})\rangle_{w}=0.04\pm 0.02$ may intuitively predict $\langle \hat{A}(t_{1})\hat{A}(t_{2})\hat{B}(t_{3})\rangle_{w}\approx 0$. Similarly, $\langle \hat{A}(t_{1})\hat{B}(t_{2})\hat{B}(t_{3})\rangle_{w}=0.54\pm 0.08$ also seems to contradict the naive prediction given by $\langle \hat{A}(t_{1})\hat{B}(t_{2})\rangle_{w}=0.17\pm 0.06$. These results indicate that it may not be possible to infer a higher order multiple-operator SWV from lower order multiple-operator SWVs. However, there is a clear connection between the lower and higher order multiple-operator SWVs via the linearity rule. In the experiment, the following relation between the double- and triple-operator SWVs can be found: $\langle \hat{A}(t_{1})\hat{A}(t_{2})\hat{B}(t_{3})\rangle_{w}+\langle \hat{A}(t_{1})\hat{B}(t_{2})\hat{B}(t_{3})\rangle_{w}+\langle \hat{A}(t_{1})\hat{C}(t_{2})\hat{B}(t_{3})\rangle_{w}=\langle \hat{A}(t_{1})(\hat{A}(t_{2})+\;\hat{B}(t_{2})+\hat{C}(t_{2}))\hat{B}(t_{3})\rangle_{w}=\langle \hat{A}(t_{1})\hat{I}\hat{B}(t_{3})\rangle_{w}=\langle \hat{A}(t_{1})\hat{B}(t_{3})\rangle_{w}$. We observe that $\langle \hat{A}(t_{1})\hat{A}(t_{2})\hat{B}(t_{3})\rangle_{w}+\langle \hat{A}(t_{1})\hat{B}(t_{2})\hat{B}(t_{3})\rangle_{w}+\langle \hat{A}(t_{1})\hat{C}(t_{2})\hat{B}(t_{3})\rangle_{w}=1.1\pm 0.1$, which is in good agreement with the theoretical prediction ($\langle \hat{A}(t_{1})\hat{B}(t_{3})\rangle_{w}=1$) and the experimental value ($\langle \hat{A}(t_{1})\hat{B}(t_{3})\rangle_{w}\;=1.00\pm 0.04$).

## Conclusion

We have experimentally explored the anomalous time evolution of a single pre- and postselected photon. The naive interpretation of the single-operator WVs seems to suggest that a photon in Mode A at $t_{1}$ disappears from Modes A and B at $t_{2}$ and re-appears in Mode B at $t_{3}$. In contrast, the double- and triple-operator SWVs show that the photon does not entirely vanish in the intermediate time. These results suggest that local values cannot fully explain the quantum time evolution—higher order correlations are, in general, necessary. Note that the nonlocal (i.e. simultaneously involving two boxes) single-operator WVs of |A⟩⟨B|+|B⟩⟨A| and i|A⟩⟨B|−i|B⟩⟨A| are theoretically not zero at $t_{2}$, which may also indicate a subtle presence of the photon in Modes A and B at $t_{2}$. It is also shown that a lower order multiple-operator SWV can be inferred by higher order multiple-operator SWVs using the linearity rule, while the opposite is impossible in general. These results provide new insights into the time evolution in quantum mechanics. The present approach may find applications in quantum technologies such as weak-value-based quantum sensing and metrology (26–32), as well as counterfactual quantum computation and communication (33–38).

## Materials and methods

### Single-operator WV measurement

Suppose we want to measure a WV of a projection operator ${\hat{\Pi }}_{k}\equiv \left|\psi_{k}\right\rangle \left\langle \psi_{k}\right|$ at a time step between two unitary transformations ${\hat{U}}_{1}$ and ${\hat{U}}_{2}$. When a quantum state $\left|\psi_{k}\right\rangle$ is attenuated, the attenuation can be represented using a nonunitary attenuation operator (28): ${\hat{L}}_{\eta_{k}}\equiv {\hat{\Pi }}_{1}+{\hat{\Pi }}_{2}+\cdots +\sqrt{\eta_{k}}{\hat{\Pi }}_{k}+\cdots +{\hat{\Pi }}_{n}$, where $\eta_{k}$ is the transmittance of $\left|\psi_{k}\right\rangle$ and $\left\{{\hat{\Pi }}_{1},{\hat{\Pi }}_{2},\ldots ,{\hat{\Pi }}_{n}\right\}$ is a complete set of projection operators that spans the *n*-dimensional Hilbert space for the system. When the postselection probability without attenuation is given by $p\equiv |\left\langle \psi_{f}\right|{\hat{U}}_{2}{\hat{U}}_{1}\left|\psi_{i}\right\rangle {|}^{2}$, the attenuation operator changes the postselection probability as follows:


(4) $$\begin{array}{rl} \;p_{L} & =|\left\langle \psi_{f}\right|{\hat{U}}_{2}{\hat{L}}_{\eta_{k}}{\hat{U}}_{1}\left|\psi_{i}\right\rangle {|}^{2}\\ & =|\left\langle \psi_{f}\right|\psi_{i}\rangle -\gamma \left\langle \psi_{f}\right|{\hat{U}}_{2}{\hat{\Pi }}_{k}{\hat{U}}_{1}\left|\psi_{i}\right\rangle {|}^{2}\\ & =p|1-\gamma \langle {\hat{\Pi }}_{k}\rangle_{w}{|}^{2}, \end{array}$$

where $\gamma \equiv 1-\sqrt{\eta_{k}}$, and $\left|\psi_{i}\right\rangle$ and $\left|\psi_{f}\right\rangle$ are the pre- and postselected states, respectively. Eq. 4 can be transformed into


(5) (Re[⟨Π^k⟩w]−1γ)2+Im[⟨Π^k⟩w]2=pLγ2p.

**Fig. 5.**
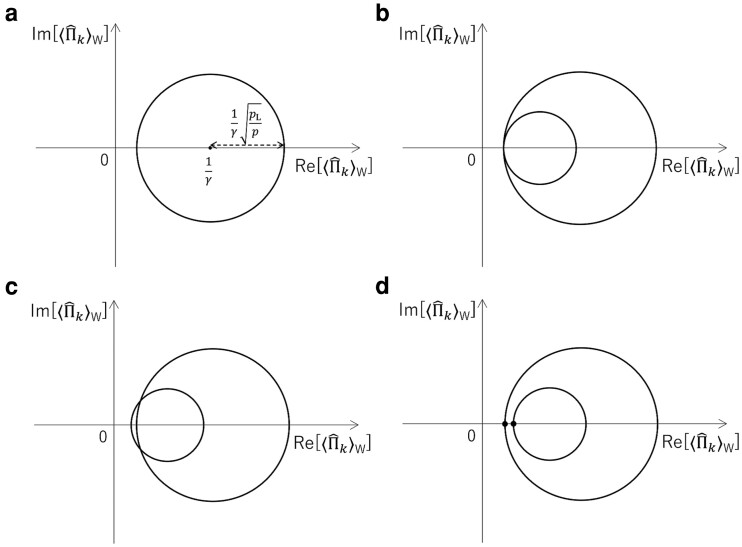
Graphical representation of the WV measurement. Eq. 5 gives the circle shown in a). The two different postselection probabilities, which are obtained with two different degrees of attenuation, give two circles with different center positions and radii. When a WV has only a real part, the two circles will have a point of contact as shown in b). When a WV includes an imaginary part, the two circles will have two intersections as shown in c). The two circles may have neither a contact nor intersections as shown in d), because of the statistical fluctuations of experimental data.

This is the equation of a circle for the variables Re[⟨Π^k⟩w] and Im[⟨Π^k⟩w] with a radius $\frac{1}{\gamma }\sqrt{\frac{\;p_{L}}{p}}$ and a center $\left(\frac{1}{\gamma },0\right)$ (see an example in Fig. 5a). Therefore, two different postselection probabilities, which are obtained with two different degrees of attenuation, are necessary to determine the WV. These postselection probabilities give two circles with different center positions and radii. When the WV has only a real part, the two circles will have a point of contact (Fig. 5b) that identifies the WV. On the other hand, when the WV includes an imaginary part, the two circles will have two intersections (Fig. 5c). In this case, while the real part and the magnitude of the imaginary part are identified, the sign of the imaginary part is indefinite. Fig. 5d shows the third case, when the two circles have neither a contact nor an intersection due to the statistical fluctuations of experimental data. To determine the WV, we seek the points on the two circles at which the distance between the two circles is the shortest. The following function is minimized:


(6) $$D({W_{R}}_{1},{W_{R}}_{2},{W_{I}}_{1},{W_{I}}_{2})=({W_{R}}_{1}-{W_{R}}_{2}{)}^{2}+({W_{I}}_{1}-{W_{I}}_{2}{)}^{2},$$

when ${W_{R}}_{1}$, ${W_{I}}_{1}$, ${W_{R}}_{2}$, and ${W_{I}}_{2}$ obey the following conditions:


(7) $$\begin{array}{rl} {\left({W_{R}}_{1}-\frac{1}{\gamma_{1}}\right)}^{2}+\;{W_{I}}_{1}^{2} & =\frac{\;p_{L1}}{\gamma_{1}^{2}p}\\ {\left({W_{R}}_{2}-\frac{1}{\gamma_{2}}\right)}^{2}+\;{W_{I}}_{2}^{2} & =\frac{\;p_{L2}}{\gamma_{2}^{2}p}, \end{array}$$

where $p_{L1}$ and $p_{L2}$ are the postselection probabilities for $\gamma_{1}$ and $\gamma_{2}$ (assumed to be $\gamma_{1}>\gamma_{2}$), respectively. If the two circles in Eq. 7 have neither a contact nor intersections, then a solution will be obtained for each circle (two dots in Fig. 5d). In this case, ${W_{R}}_{1}$ and ${W_{I}}_{1}$ are adopted because a larger γ results in a smaller radius of the circle and a smaller effect of the fluctuations in the experimental values ($p_{L}$, *p*). Note that although the sign of the imaginary part cannot be determined in this scheme, the magnitude of the imaginary part is expected to be very small in this experiment because all theoretically predicted WVs in DRP have only real parts.

In order to estimate the single-operator WVs using this probeless scheme, we experimentally obtained the values of *p*, $p_{L1}$, $p_{L2}$, $\gamma_{1}$, and $\gamma_{2}$ in Eq. 7. As defined above, *p* characterizes the postselection probability without an attenuator. Therefore, it was obtained by measuring the number of postselected photons with the setup shown in Fig. 2a which can be represented as $N=N_{0}p$, where $N_{0}$ is the output photon number without postselection. Since $p_{L1}$ characterizes the postselection probability when an attenuator with transmittance of $(1-\gamma_{1}{)}^{2}$ is placed at the location corresponding to the target WV, it was obtained by measuring the number of postselected photons after the attenuator insertion. This measured photon number can be represented as $N_{L1}=N_{0}p_{L1}$. Then, the value of $p_{L2}$ was obtained in a similar way, but with an attenuator that has a different transmittance (represented as $(1-\gamma_{2}{)}^{2}$) placed at the same location. The values of $\gamma_{1}$ and $\gamma_{2}$ were precisely estimated through independent transmittance measurements of the attenuators. These experimentally obtained values were substituted into Eq. 7, and then minimizing Eq. 6 provided the single-operator WVs shown in Fig. 3.

### Double-operator SWV measurement

Suppose we want to measure an SWV of two projection operators: ${\hat{\Pi }}_{k}\equiv \left|\psi_{k}\right\rangle \left\langle \psi_{k}\right|$ at a time step between two unitary transformations ${\hat{U}}_{1}$ and ${\hat{U}}_{2}$, and ${\hat{\Pi }}_{l}\equiv \left|\psi_{l}\right\rangle \left\langle \psi_{l}\right|$ at a time step between two unitary transformations ${\hat{U}}_{2}$ and ${\hat{U}}_{3}$. When the attenuation operators ${\hat{L}}_{\eta_{k}}$ and ${\hat{L}}_{\eta_{l}}$ are applied between ${\hat{U}}_{1}$ and ${\hat{U}}_{2}$, ${\hat{U}}_{2}$, and ${\hat{U}}_{3}$, respectively, the postselection probability changes to


(8) $$\begin{array}{rl} \;p_{\mathrm{DL}} & =|\left\langle \psi_{f}\right|{\hat{U}}_{3}{\hat{L}}_{\eta_{l}}{\hat{U}}_{2}{\hat{L}}_{\eta_{k}}{\hat{U}}_{1}\left|\psi_{i}\right\rangle {|}^{2}\\ & =p|X-\gamma_{k}\gamma_{l}\langle {\hat{\Pi }}_{k}(t_{1}){\hat{\Pi }}_{l}(t_{2})\rangle_{w}{|}^{2}, \end{array}$$

where $X\equiv \gamma_{k}\langle {\hat{\Pi }}_{k}(t_{1})\rangle_{w}+\gamma_{l}\langle {\hat{\Pi }}_{l}(t_{2})\rangle_{w}-1$, $\gamma_{k}\equiv 1-\sqrt{\eta_{k}}$, and $\gamma_{l}\equiv 1-\sqrt{\eta_{l}}$. Eq. 8 can be transformed into


(9) (Re[⟨Π^k(t1)Π^l(t2)⟩w]−Re[X]γkγl)2+(Im[⟨Π^k(t1)Π^l(t2)⟩w]−Im[X]γkγl)2=pDLγk2γl2p.

This is the equation of a circle for the variables Re[⟨Π^k(t1)Π^l(t2)⟩w] and Im[⟨Π^k(t1)Π^l(t2)⟩w] with a radius $\frac{1}{\gamma_{k}\gamma_{l}}\sqrt{\frac{\;p_{\mathrm{DL}}}{p}}$ and a center (Re[X]γkγl,Im[X]γkγl). Similarly to the case of the single-operator WV measurement, two different postselection probabilities, which are obtained with two different combinations of attenuators, are necessary to determine the SWV. These two measurement results give two circles with different center positions and radii. The following function is then minimized to seek the points on the two circles where the distance between the two circles is the shortest:


(10) $$D({W_{R}}_{1},{W_{R}}_{2},{W_{I}}_{1},{W_{I}}_{2})=({W_{R}}_{1}-{W_{R}}_{2}{)}^{2}+({W_{I}}_{1}-{W_{I}}_{2}{)}^{2},$$

when ${W_{R}}_{1}$, ${W_{I}}_{1}$, ${W_{R}}_{2}$, and ${W_{I}}_{2}$ obey the following conditions:


(11) (WR1−Re[X]γk1γl1)2+(WI1−Im[X]γk1γl1)2=pDL1γk12γl12p,(WR2−Re[X]γk2γl2)2+(WI2−Im[X]γk2γl2)2=pDL2γk22γl22p,

where $p_{\mathrm{DL}1}$ is the postselection probability for $\gamma_{k1}$ and $\gamma_{l1}$, and $p_{\mathrm{DL}2}$ is the postselection probability for $\gamma_{k2}$ and $\gamma_{l2}$. We assume that $\gamma_{k1}\gamma_{l1}>\gamma_{k2}\gamma_{l2}$. The values of $\langle {\hat{\Pi }}_{k}(t_{1})\rangle_{w}$ and $\langle {\hat{\Pi }}_{l}(t_{2})\rangle_{w}$ obtained in the single-operator WV measurement are substituted to determine *X*. The sign of the imaginary part of the single-operator WV is undetermined in single-operator WV measurement; therefore, the combination of the signs that gives the shortest distance is adopted. Note that in the experiment, the obtained double-operator SWVs are not significantly dependent on the signs of the imaginary parts of the single-operator WVs, because of their small magnitudes. If the two circles in Eq. 11 have neither a contact nor intersections due to statistical fluctuations of experimental data, then two solutions will be obtained for each circle. In this case, ${W_{R}}_{1}$ and ${W_{I}}_{1}$ are adopted for a similar reason to that for single-operator WV measurement.

In order to estimate the double-operator SWVs using this probeless scheme, we experimentally obtained the values of *p*, $p_{\mathrm{DL}1}$, $p_{\mathrm{DL}2}$, $\gamma_{k1}$, $\gamma_{l1}$, $\gamma_{k2}$, $\gamma_{l2}$, and *X* in Eq. 11. The value of *p* was obtained in the same way used for the case of the single-operator WV measurement. The value of $p_{\mathrm{DL}1}$ characterizes the postselection probability when two attenuators are placed at the two locations corresponding to the target SWV. For example, in order to obtain $p_{\mathrm{DL}1}$ for $\langle \hat{A}(t_{1})\hat{C}(t_{2})\rangle_{w}$, we inserted one of two attenuators into Mode A at $t_{1}$ and the other into Mode C at $t_{2}$ as shown in Fig. 2b. The transmittances of the two attenuators are represented as $(1-\gamma_{k1}{)}^{2}$ and $(1-\gamma_{l1}{)}^{2}$ using the above definition. Note that in our experiment the same transmittance is adopted for both attenuators so that $\gamma_{k1}=\gamma_{l1}$. Then, the value of $p_{\mathrm{DL}2}$ was obtained in a similar way, but with attenuators that have different transmittances (represented as $(1-\gamma_{k2}{)}^{2}$ and $(1-\gamma_{l2}{)}^{2}$) placed at the same locations. The values of $\gamma_{k1}$, $\gamma_{l1}$, $\gamma_{k2}$, and $\gamma_{l2}$ were precisely estimated through independent transmittance measurements of the attenuators. To obtain the value of *X*, the single-operator WVs corresponding to the target double-operator SWV must be measured beforehand. For example, when the target double-operator SWV is $\langle \hat{A}(t_{1})\hat{C}(t_{2})\rangle_{w}$, the corresponding single-operator WVs are $\langle \hat{A}(t_{1})\rangle_{w}$ and $\langle \hat{C}(t_{2})\rangle_{w}$. These experimentally obtained values were substituted into Eq. 11, and then minimizing Eq. 10 provided the double-operator SWVs shown in Fig. 4a and b.

### Triple-operator SWV measurement

Suppose that we want to obtain an SWV of three projection operators: ${\hat{\Pi }}_{k}$ at a time step between the unitary transformations ${\hat{U}}_{1}$ and ${\hat{U}}_{2}$, ${\hat{\Pi }}_{l}$ at a time step between the unitary transformations ${\hat{U}}_{2}$ and ${\hat{U}}_{3}$, and ${\hat{\Pi }}_{m}$ at a time step between the unitary transformations ${\hat{U}}_{3}$ and ${\hat{U}}_{4}$. When the attenuation operators ${\hat{L}}_{\eta_{k}}$${\hat{L}}_{\eta_{l}}$ and ${\hat{L}}_{\eta_{m}}$ are applied between ${\hat{U}}_{1}$ and ${\hat{U}}_{2}$, ${\hat{U}}_{2}$ and ${\hat{U}}_{3}$, and ${\hat{U}}_{3}$ and ${\hat{U}}_{4}$, respectively, the postselection probability changes to


(12) $$\begin{array}{rl} \;p_{\mathrm{TL}} & =|\left\langle \psi_{f}\right|{\hat{U}}_{4}{\hat{L}}_{\eta_{m}}{\hat{U}}_{3}{\hat{L}}_{\eta_{l}}{\hat{U}}_{2}{\hat{L}}_{\eta_{k}}{\hat{U}}_{1}\left|\psi_{i}\right\rangle {|}^{2}\\ & =p|Y-\gamma_{k}\gamma_{l}\gamma_{m}\langle {\hat{\Pi }}_{k}(t_{1}){\hat{\Pi }}_{l}(t_{2}){\hat{\Pi }}_{m}(t_{3})\rangle_{w}{|}^{2}, \end{array}$$

where $Y\equiv 1-\gamma_{k}\langle {\hat{\Pi }}_{k}(t_{1})\rangle_{w}-\;\gamma_{l}\langle {\hat{\Pi }}_{l}(t_{2})\rangle_{w}-\gamma_{m}\langle {\hat{\Pi }}_{m}(t_{3})\rangle_{w}\;\;+\;\;\gamma_{k}\gamma_{l}\langle {\hat{\Pi }}_{k}(t_{1}){\hat{\Pi }}_{l}(t_{2})\rangle_{w}\;+\;\;\;\gamma_{k}\gamma_{m}\langle {\hat{\Pi }}_{k}(t_{1}){\hat{\Pi }}_{m}(t_{3})\rangle_{w}\;\;+\;\;\gamma_{l}\gamma_{m}\langle {\hat{\Pi }}_{l}(t_{2}){\hat{\Pi }}_{m}(t_{3})\rangle_{w}$, $\gamma_{k}\equiv 1\;-\sqrt{\eta_{k}}$, $\gamma_{l}\equiv 1\;-\sqrt{\eta_{l}}$, and $\gamma_{m}\equiv 1-\sqrt{\eta_{m}}$. Eq. 12 can be transformed into


(13) (Re[⟨Π^k(t1)Π^l(t2)Π^m(t3)⟩w]−Re[Y]γkγlγm)2+(Im[⟨Π^k(t1)Π^l(t2)Π^m(t3)⟩w]−Im[Y]γkγlγm)2=pTLγk2γl2γm2p.

This is the equation of a circle for the variables Re[⟨Π^k(t1)Π^l(t2)Π^m(t3)⟩w] and Im[⟨Π^k(t1)Π^l(t2)Π^m(t3)⟩w] with a radius $\frac{1}{\gamma_{k}\gamma_{l}\gamma_{m}}\sqrt{\frac{\;p_{\mathrm{TL}}}{p}}$ and a center (Re[Y]γkγlγm,Im[Y]γkγlγm). Similar to the case of the single-operator WV measurement, two different postselection probabilities, which are obtained with two different combinations of attenuators, are necessary to determine the SWV. These two measurement results give two circles with different center positions and radii. The following function is then minimized to seek the points on the two circles where the distance between the two circles is the shortest:


(14) $$D({W_{R}}_{1},{W_{R}}_{2},{W_{I}}_{1},{W_{I}}_{2})\;=\;({W_{R}}_{1}-{W_{R}}_{2}{)}^{2}\;+\;({W_{I}}_{1}-{W_{I}}_{2}{)}^{2},$$

when ${W_{R}}_{1}$, ${W_{I}}_{1}$, ${W_{R}}_{2}$, and ${W_{I}}_{2}$ obey the following conditions:


(15) (WR1−Re[Y]γk1γl1γm1)2+(WI1−Im[Y]γk1γl1γm1)2=pTL1γk12γl12γm12p,(WR2−Re[Y]γk2γl2γm2)2+(WI2−Im[Y]γk2γl2γm2)2=pTL2γk22γl22γm22p,

where $p_{\mathrm{TL}1}$ is the postselection probability for $\gamma_{k1}$, $\gamma_{l1}$, and $\gamma_{m1}$, and $p_{\mathrm{TL}2}$ is the postselection probability for $\gamma_{k2}$, $\gamma_{l2}$, and $\gamma_{m2}$. We assume that $\gamma_{k1}\gamma_{l1}\gamma_{m1}>\gamma_{k2}\gamma_{l2}\gamma_{m2}$. The obtained single-operator WVs and double-operator SWVs are substituted to determine *Y*. If the two circles in Eq. 15 have neither a contact nor intersections due to statistical fluctuations of experimental data, then two solutions will be obtained for each circle. In this case, ${W_{R}}_{1}$ and ${W_{I}}_{1}$ are adopted for a similar reason to that for single-operator WV measurement.

It is straightforward to extend this scheme to an arbitrary number of operators. Note that it has also been shown that the imaginary part of a WV can be measured using a small phase shift without a probe (39, 20). Therefore, using an approach similar to that shown here, it may be possible to apply it for an arbitrary magnitude of phase to find the sign of the imaginary parts of WVs (although this was not required for the current analysis).

In order to estimate the triple-operator SWVs using this probeless scheme, we experimentally obtained the values of *p*, $p_{\mathrm{TL}1}$, $p_{\mathrm{TL}2}$, $\gamma_{k1}$, $\gamma_{l1}$, $\gamma_{m1}$, $\gamma_{k2}$, $\gamma_{l2}$, $\gamma_{m2}$, and *Y* in Eq. 15. The value of *p* was obtained in the same way used for the case of the single-operator WV measurement. The value of $p_{\mathrm{TL}1}$ characterizes the postselection probability when three attenuators are placed at the three locations corresponding to the target SWV. For example, in order to obtain $p_{\mathrm{TL}1}$ for $\langle \hat{A}(t_{1})\hat{C}(t_{2})\hat{B}(t_{3})\rangle_{w}$, we inserted one of three attenuators into Mode A at $t_{1}$, another into Mode C at $t_{2}$ and the other into Mode B at $t_{3}$. The transmittances of the three attenuators are represented as $(1-\gamma_{k1}{)}^{2}$, $(1-\gamma_{l1}{)}^{2}$, and $(1-\gamma_{m1}{)}^{2}$ using the above definition. Note that in our experiment the same transmittance is adopted for these attenuators so that $\gamma_{k1}=\gamma_{l1}=\gamma_{m1}$. Then, the value of $p_{\mathrm{TL}2}$ was obtained in a similar way, but with attenuators that have different transmittances (represented as $(1-\gamma_{k2}{)}^{2}$, $(1-\gamma_{l2}{)}^{2}$, and $(1-\gamma_{m2}{)}^{2}$) placed at the same locations. The values of $\gamma_{k1}$, $\gamma_{l1}$, $\gamma_{m1}$, $\gamma_{k2}$, $\gamma_{l2}$, and $\gamma_{m2}$ were precisely estimated through independent transmittance measurements of the attenuators. To obtain the value of *Y*, the single-operator WVs and double-operator SWVs corresponding to the target triple-operator SWV must be mesured beforehand. For example, when the target triple-operator SWV is $\langle \hat{A}(t_{1})\hat{C}(t_{2})\hat{B}(t_{3})\rangle_{w}$, $\langle \hat{A}(t_{1})\rangle_{w}$, $\langle \hat{C}(t_{2})\rangle_{w}$, and $\langle \hat{B}(t_{3})\rangle_{w}$ are the corresponding single-operator WVs and $\langle \hat{A}(t_{1})\hat{C}(t_{2})\rangle_{w}$, $\langle \hat{A}(t_{1})\hat{B}(t_{3})\rangle_{w}$, and $\langle \hat{C}(t_{2})\hat{B}(t_{3})\rangle_{w}$ are the corresponding double-operator SWVs. These experimentally obtained values were substituted into Eq. 15, and then minimizing Eq. 14 provided the triple-operator SWVs shown in Fig. 4c.

## Data Availability

All study data are included in the article.
